# New insights into the impact of neuro-inflammation in rheumatoid arthritis

**DOI:** 10.3389/fnins.2014.00357

**Published:** 2014-11-06

**Authors:** Nicholas R. Fuggle, Franklyn A. Howe, Rachel L. Allen, Nidhi Sofat

**Affiliations:** ^1^Institute of Infection and Immunity, St. George's UniversityLondon, UK; ^2^Neuroscience Research Centre, Institute of Cardiovascular and Cell Sciences, St. George's UniversityLondon, UK

**Keywords:** neuroinflammation, rheumatoid arthritis, tumor necrosis factor alpha, neuroimaging, infection, lipopolysaccharide

## Abstract

Rheumatoid arthritis (RA) is considered to be, in many respects, an archetypal autoimmune disease that causes activation of pro-inflammatory pathways resulting in joint and systemic inflammation. RA remains a major clinical problem with the development of several new therapies targeted at cytokine inhibition in recent years. In RA, biologic therapies targeted at inhibition of tumor necrosis factor alpha (TNFα) have been shown to reduce joint inflammation, limit erosive change, reduce disability and improve quality of life. The cytokine TNFα has a central role in systemic RA inflammation and has also been shown to have pro-inflammatory effects in the brain. Emerging data suggests there is an important bidirectional communication between the brain and immune system in inflammatory conditions like RA. Recent work has shown how TNF inhibitor therapy in people with RA is protective for Alzheimer's disease. Functional MRI studies to measure brain activation in people with RA to stimulus by finger joint compression, have also shown that those who responded to TNF inhibition showed a significantly greater activation volume in thalamic, limbic, and associative areas of the brain than non-responders. Infections are the main risk of therapies with biologic drugs and infections have been shown to be related to disease flares in RA. Recent basic science data has also emerged suggesting that bacterial components including lipopolysaccharide induce pain by directly activating sensory neurons that modulate inflammation, a previously unsuspected role for the nervous system in host-pathogen interactions. In this review, we discuss the current evidence for neuro-inflammation as an important factor that impacts on disease persistence and pain in RA.

## Introduction

Rheumatic diseases include some of the most common chronic disorders worldwide. Of these, rheumatoid arthritis (RA) is considered to be, in many respects, an archetypal autoimmune disease (Feldmann and Maini, 2010). RA causes activation of pro-inflammatory pathways resulting in joint and systemic inflammation and remains a major clinical problem (McInnes and Schett, 2011). Treatments with immune-modulatory drugs, including biologic therapies, have revolutionized its management. New therapies target relevant cytokines such as tumor necrosis factor alpha (TNFα) and immune cells such as B cells. In RA, biologics have been shown to reduce joint inflammation, limit erosive change, reduce disability and improve quality of life (Scott et al., 2010). Biologics are mainly co-administered with disease-modifying drugs such as methotrexate when the latter are found to achieve insufficient disease control on their own (Firestein, 2003). Recent work using functional neuroimaging has suggested that TNF inhibitors may also reduce central nervous system activity related to inflammation-induced pain in people with RA using brain neuroimaging (Rech et al., 2013). Classically, the term “neuro-inflammation” has implicated the activation of microglia and astroglia which in turn activate the expression of pro-inflammatory cytokines and chemokines in a variety of conditions including Alzheimer's disease (AD) (O'Callaghan et al., 2008). However, the trigger(s) for activation of a potential neuro-inflammatory process and furthermore its measurement in clinical disease is not always straightforward to decipher. Several groups have utilized animal models to discover that although certain features of inflammation are typical for the pro-inflammatory response systemically e.g., interleukin-6 (IL-6), interferon-β (IFNβ), these are not observed in the brain, but activation of TNFα does appear to maintain pro-inflammatory activity in the brain as it does systemically (Skelly et al., 2013; Thomson et al., 2014). Some of the basic science observations can be extended to RA, where Chou et al. (2010) observed that people with RA receiving TNF inhibitor treatment (infliximab, etanercept, adalimumab) showed a reduction in the risk of developing AD compared to controls (Chou et al., 2010). The risk of AD was not affected by exposure to other Disease-Modifying Anti-Rheumatic Drugs (DMARDs) used in RA. Recent work by Detrait et al. (2014) has also shown that peripheral administration of TNF inhibitors in mice counteracts the amyloid-induced increase of hippocampal TNFα levels and memory deficits in mice. In this review, we explore the theme of neuro-inflammation in RA. Although as yet, the cause-and-effect relationship between infection, autoimmunity, neural correlates and RA disease activity are not understood mechanistically, we review the current status of the field.

## Current concepts in the pathogenesis of rheumatoid arthritis

RA is an immune-mediated pro-inflammatory disease, often resulting in chronic disability, early mortality, systemic complications and high socioeconomic burden on society as a whole (McInnes and Schett, 2011). There has been a vast improvement in our understanding of this condition over the last few decades, resulting in improved treatments which are now initiated early in order to maximize and maintain disease remission (van Vollenhoven, 2010).

Risks for developing RA involve a complex interplay between genotype, environment and lifestyle factors such as smoking (Mahdi et al., 2009). Twin studies have shown a concordance rate of 15–30% among monozygotic twins and 5% among dizygotic twins (MacGregor et al., 2000). Genome-wide association analyses have uncovered immune regulatory factors that may underlie the disease; including PTPN22 among the Single Nucleotide Polymorphisms (SNPs) identified (Wellcome Trust Case Control Consortium, 2007). An association with HLA-DRB1 has been established for RA patients who are positive for rheumatoid factor or anti-citrullinated peptide antibodies (ACPA) (Gregersen et al., 1987). In keeping with the role of HLA-DRB1 in antigen presentation, a number of studies over the last 2 decades have shown that auto-reactive immune responses are mediated by T-cell repertoire selection, antigen presentation or changes in peptide affinity (Panayi, 2006). The shared epitope (SE), carried by the vast majority of RA patients, is a 5-aa sequence motif in the third allelic hypervariable region of the HLA-DRβ chain. Proposed explanations for the link between RA and the SE include molecular mimicry of the SE by microbial proteins, increased T cell senescence induced by SE-containing HLA molecules and a potential pro-inflammatory signaling function that is unrelated to the role of the SE in antigen recognition (Weyand and Goronzy, 1990; De Almeida et al., 2010).

Gene-environment interactions are also important in RA development. Smoking and other environmental risks to the lung such as silica exposure, increase the risk of RA in people with susceptibility HLA-DR4 alleles (Symmons et al., 1997; Klareskog et al., 2008). Smoking and HLA-DRB1 alleles synergistically increase one's risk of developing the anti-citrullinated protein antibodies (ACPA) that are present in the majority of patients with RA (Klareskog et al., 2008). It has therefore been proposed that environmental stress in the lung or other mucosal surfaces may promote post-translational modifications through activation of peptidyl arginine deiminase, type IV (PADIV), resulting in citrullination of mucosal proteins. Loss of tolerance to the neoepitopes generated by citrullination can be detected clinically in people with RA by the ACPA response (Vincent et al., 1999).

It has long been recognized that infectious agents such as *cytomegalovirus, E. coli, Epstein Barr virus, parvovirus*, and *proteus* species may play a role in RA. Recently, the oral pathogen *Porphyromonas gingivalis* has been implicated in the pathogenesis of RA (Mikuls et al., 2014). Products of infectious agents e.g., heat shock proteins and enzymes responsible for citrullination have been shown in several models to induce immune reactivity. For example, several citrullinated self-proteins can be identified in ACPA assays, including alpha enolase, keratin, fibrinogen, fibronectin, collagen, and vimentin (van der Woude et al., 2010). Although unifying mechanisms for the link between infection and RA autoimmunity are not entirely established, the theory of molecular mimicry has been proposed (van Heemst et al., 2014). The formation of immune complexes during infection may trigger the induction of rheumatoid factor, which is a high affinity autoantibody against the Fc portion of immunoglobulin, often used in the diagnosis of RA (De Rycke et al., 2004). A link has been described between RA and periodontal disease: *Porphyromonas gingivalis* produces PAD1V which can promote citrullination of mammalian proteins (Wegner et al., 2010). Recently, the gastrointestinal microbiome has also been implicated in the development of autoimmunity (Scher et al., 2012).

Emerging data suggests there is an important impact of bidirectional communication between the brain and immune system that has a significant impact on RA symptoms. The onset of RA is associated with adverse life events and infectious triggers, where links between the hypothalamic-pituitary-adrenal axis and autoimmunity have been shown (Sokka et al., 2009; Capellino et al., 2010). The impact of external factors such as infections and their impact on disease activity in RA including joint pain/swelling will be discussed further in the following sections. One feature of established RA includes the presence of rheumatoid factor, which is an IgM complex of antibodies to IgG found circulating in RA (Mellor, 1959). Cerebrospinal fluid rheumatoid factor has been demonstrated in case reports of central nervous system manifestations of RA. (Markenson et al., 1979; Inan et al., 2011) Recent studies suggest that circulating immune complexes can elicit a neuro-inflammatory response in the brain. Teeling et al. (2012) showed that in the presence of antigen, antibodies can lead to local immune complex-mediated inflammatory reaction in the brain parenchyma and directly induce local tissue damage through the recruitment and activation of microglia through Fcγ receptors (Teeling et al., 2012).

## The role of cyclo-oxygenase and prostanoids in neuro-inflammation

Several of the brain effects induced by cytokines such as TNFα are thought to be mediated by regulating the expression of cyclo-oxygenase (COX) enzymes (mainly COX-2) and generation of prostanoids. Under usual physiological conditions, these inflammatory mediators are absent in the brain or found in low levels. After brain injury or insult, there can be induction of pro-inflammatory mediators in astrocytes, microglia, neurons and endothelial cells which results in the development of neuro-inflammatory conditions. Recently, the COX-2 inhibitor celecoxib has been shown to reduce functional connectivity measured by functional MRI in an animal model of osteoarthritis (Upadhyay et al., 2013). In their study, Upadhyay et al. (2013) showed a reduction in blood oxygenation in recognized brain pain centers including the thalamus, hippocampus, periaqueductal gray matter and nucleus accumbens in arthritic rodents treated with celecoxib. Human studies in patients with chronic knee osteoarthritis have also shown that COX inhibitors e.g., valdecoxib, reduce spontaneous brain pain activation signals measured by functional brain MRI (Parks et al., 2011).

One of the earliest studies in human subjects by Ho et al. (2001) demonstrated that neuronal COX-2 expression was increased in post-mortem samples of people with Alzheimer's disease and correlated with clinical progression of dementia (Ho et al., 2001). In Alzheimer's disease, the classical neuro-pathological changes include deposition of neurofibrillary tangles and β amyloid (Aβ). Alzheimer's dementia is typified by activation of microglia and astrocytes in response to Aβ deposition, leading to a release of a variety of factors including the cytokines TNFα and IL-1β, free radicals such as nitric oxide, superoxide and cyclo-oxygenase pathway derived prostanoids (Combs et al., 2001; Hull et al., 2006; Medeiros et al., 2007). Evidence for the activation of the COX pathway has come from neuro-inflammatory diseases that include Alzheimer's disease, Parkinson's disease and multiple sclerosis. For example, in a mouse model of Parkinson's disease, Hunter et al. (2007) showed that celecoxib, a COX-2 inhibitor, inhibited neuronal effects in rats injected with LPS. They found that a number of effects were reduced in rats treated with celecoxib, including inflammation and nigral dopaminergic neuronal loss. It is therefore possible that non-steroidal anti-inflammatory drugs (NSAIDs) such as celecoxib could have an inhibitory effect on neuro-inflammation and could have an impact on delaying neurodegenerative effects.

## Can infection influence rheumatoid arthritis pain?

The central nervous system is involved in immune regulation and homeostasis, with neuro-immunological interactions regulating disease development in animal models of arthritis (Chiu et al., 2013). Peripheral inflammation triggered by synovial inflammation in RA stimulates the increased release of cytokines (Taylor, 2014). Cytokine inhibitors administered to patients with a high disease load have also been shown to suppress RA arthritic disease activity (Feldmann and Maini, 2010). More recently, microarray analysis of brain extracts from mice injected peripherally with the gram-negative bacterial stimulus of lipopolysaccharide (LPS) has indicated an increase in cytokine expression (Thomson et al., 2014). The cytokine up regulation observed was found to be elicited by a Toll-Like Receptor (TLR)-mediated interferon response in mice injected peripherally with LPS (Thomson et al., 2014).

Data has emerged from animal models to suggest that microbial components including LPS can activate sensory neurons directly (Chiu et al., 2013). In their elegant studies, Chiu et al. (2013) applied heat-killed *S. aureus* to dorsal root ganglia (DRG) sensory neurons and demonstrated an induction of calcium flux and induced action potential firing. *S. aureus* was found to directly activate nociceptors in the Nav1.8-lineage (Chiu et al., 2013). Other experiments showed that neuronal responses by the Nav 1.8 nociceptor could be activated through heat-killed *streptococcus*, *Listeria monocytogenes, Mycoplasma fermentans, Helicobacter pylori, Pseudomonas aeruginosa*, and *Escherichia coli*. Variations in the pattern of nociceptor responsiveness between bacteria could indicate that strain-specific ligands act through different mechanisms.

## The importance of TNF as a pro-inflammatory cytokine in rheumatoid arthritis

Biologic therapies have been used to treat severe RA since 1997 and have been manufactured to target specific elements of inflammation pathways. They include anakinra, an IL-1 antagonist; abatacept, which down-regulates T cell activation; rituximab, a chimeric anti-CD20 antibody which reduces B cell activation and infliximab, adalimumab and etanercept which inhibit TNF activity (Malaviya and Ostor, 2012). The European League Against Rheumatism (EULAR) and American College of Rheumatology (ACR) provide guidance on suitable indications for commencing biologic therapy for RA recommending treatment for disease which is active despite optimization of conventional DMARDs or for patients with high disease activity and risk factors for poor outcome (ACPA and rheumatoid factor positivity and erosive disease on radiography) (Smolen et al., 2014).

As mentioned above, TNFα is a key cytokine in the pathogenesis of synovial inflammation in RA, however, it has also been shown to play a vital role in fighting infections in animal models (Parks et al., 2011). TNFα is a type II transmembrane protein, cleaved by TNF-α converting enzyme (TACE) to a soluble form. It acts as a ligand for two receptors, TNFR1 and TNFR2, to enable transduction of anti-apoptotic, pro-inflammatory signals. TNFα-mediated functions include phagosome maturation, autophagy inhibition and apoptosis following activation of caspase 8 by TNFR1 (Harris and Keane, 2010). By inhibiting these actions, anti-TNF therapy causes suppression of the immune system (Tracey et al., 2008) and improves outcomes for suppressing inflammation and function clinically. However, TNF inhibitor therapy has also been shown to induce neurological events (Kaltsonoudis et al., 2014).

## Types of infections

The risk of developing infections is heightened in RA. In the following sections we describe the more common infections observed in people with RA. Since TNF inhibitor therapy can increase the incidence of infections in RA, it is possible that increased infections observed in RA during TNF inhibitor therapy could be a cause of RA flares, which in turn could have an impact on RA disease activity.

### Bacterial infections

Bacterial infections contribute to three quarters of infections in patients taking corticosteroids (CS), DMARDs or biological agents for RA and spondyloarthritis with respiratory tract infections being the most common (Germano et al., 2014). A recent systematic review of over 23,000 patients using anti-TNF therapy in the treatment of RA, juvenile idiopathic arthritis, ankylosing spondylitis, psoriatic arthritis, psoriasis and Crohn's disease, has further characterized infections associated with biologics. It showed that infections leading to a cessation in treatment included pneumonia (6.6%), bacterial arthritis (2.8%), gastro-intestinal abscess (2.1%), and cellulitis (1.1%). In this study the highest rates of serious infection events were seen in RA and, within this subset of patients, cellulitis and pneumonia were the most common types of infection (Burmester et al., 2013).

### Mycobacterial infections

TNFα is a key mediator in the host immune defense against mycobacterium tubercule bacillus (TB) infections leading to activation of macrophages, cell recruitment, granuloma formation and maintenance (Bean et al., 1999). In murine models, the neutralization of TNFα increases susceptibility to primary TB and injection of soluble TNFα receptors can cause activation of TB in infected mice (Senaldi et al., 1996). TNF inhibitor therapy may also result in active tuberculosis in humans carrying a latent infection (Keane et al., 2001). A 3 year study by Tubach (Tubach et al., 2009) found 69 cases of tuberculosis (40 in RA) in patients treated with anti-TNFα. Tuberculosis has been found to occur more frequently in patients taking anti-TNFα therapy than in the general population with a standardized incidence rate varying between 12 and 35 (Ramiro et al., 2014). There is data to suggest that the risk of tuberculosis is not constant for all anti-TNFα medications, with soluble TNFα receptors resulting in a lower risk of infection than monoclonal antibodies (Tubach et al., 2009).

### Viral and opportunistic infections

Some studies separate opportunistic infections from a broader definition of infections, serious infections, or serious infection events. In a study of 23, 458 patients on anti-TNFα therapy 20 patients (14 patients with RA) developed opportunistic infections (excluding TB or oral candidiasis) including oesophageal candidiasis, aspergillosis, candida sepsis, coccidiomycosis, cytomegalovirus, herpes zoster, and nocardiasis (Burmester et al., 2013).

Analysis of a French registry for opportunistic infections in patients taking biologic therapy for any condition found that 43 patients had opportunistic infections; 29 on infliximab, 10 on adalimumab and 4 on etanercept (Salmon-Ceron et al., 2011). Twenty-six of these were experienced by patients with RA and, of the total 43, 10 patients required intensive care and four died. When interpreting these findings it should be noted that patient information included in registry data is unlikely to be subject to the close scrutiny and follow-up afforded as those from clinical trials. Thus, systematic reviews of randomized controlled trials and open-label trials may provide more robust data.

The risk of herpes zoster infection is higher in those with autoimmune conditions than in the general population (Wolfe et al., 2006) and rates of the disease are significantly increased for those receiving anti-TNF therapy (and monoclonal antibodies in particular), with an increased rate with greater age and higher disease activity at baseline (Strangfeld et al., 2009). However, a more recent systematic review including three studies investigating skin infections in patients taking biologic therapies calculated an adjusted hazard ratio range of 1.0–1.7 for herpes zoster infection, reflecting no significant risk of the infection when taking biological DMARDs (Ramiro et al., 2014).

## Risk of infection

Randomized controlled trials are mixed in their demonstration of an association between biologic therapy and infectious adverse events (Lipsky et al., 2000; Keystone et al., 2004; Wallis et al., 2004). It has been demonstrated that of the patients with RA who acquire serious infections whilst taking biologics, 32% permanently discontinue therapy (Burmester et al., 2013) leading to significant compromise in therapeutic options for the treatment of the patient's arthritis.

The higher rate of infectious events may result from an increased risk of infection inherent to RA (Doran et al., 2002a) (though this appears to have reduced over the last 50 years (Ni Mhuircheartaigh et al., 2013), which is thought to be secondary to immune disturbance associated with disease pathogenesis and the use of immune-modulators to control the condition (Doran et al., 2002a,b). An inherent risk of infection makes establishing a causal link between biologic DMARDs and infections more difficult. For this reason systematic reviews and meta-analyses of clinical trials and registry data are useful tools to investigate the rare adverse events, by pooling data for large cohorts of patients.

**Figure 1:**
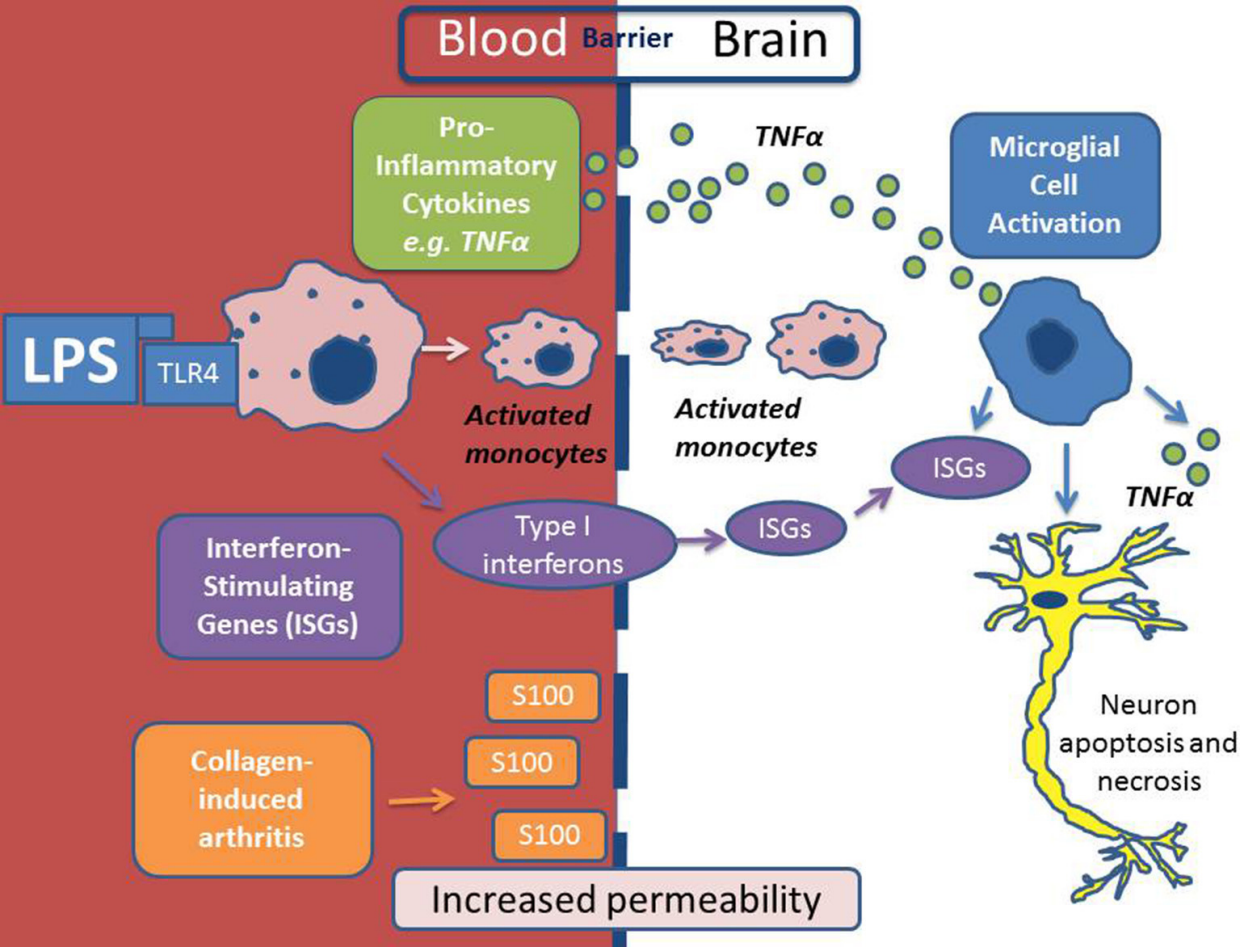
**Scheme depicting the mechanism of neuro-inflammation in autoimmune conditions**. In this depiction lipopolysaccharide (LPS) is the inflammatory agent which causes activation of monocytes in the systemic circulation via Toll-Like Receptor 4 (TLR4). LPS/TLR4 activation results in the production of pro-inflammatory cytokines (including Tumor Necrosis Factor alpha (TNFα) and Interferon Stimulating Genes (ISGs) which activate microglial cells within the brain. Activated monocytes are then recruited across the BBB and as a result of all of the above there may be consequential neuronal apoptosis and necrosis. In a model of collagen-induced arthritis, S100 was shown to increase BBB permeability to inflammatory cells.

Recent work has suggested that the blood brain barrier (BBB) may be compromised in RA with macrophages being recruited from the systemic circulation via intercellular adhesion molecule (ICAM) expression on the endothelium (Jacobs and Tavitian, 2012). Increased cerebrovascular permeability has been demonstrated in mice challenged with the collagen-induced arthritis (CIA) model (Nishioku et al., 2011). The same group have also proposed that the S100A4 protein mediates disruption of the BBB by increasing vascular permeability (Nishioku et al., 2011). Further evidence that the BBB is not as immunologically a privileged site as previously thought is supported by the recent observations in mice with systemic inflammation triggered by a peripheral LPS challenge show activation of interferon-stimulated genes (ISGs) and TNFα in the brain (Thomson et al., 2014), as illustrated in Figure 1. Chronic inflammatory diseases, including RA, are observationally associated with neuropsychiatric features such as depression, anxiety, pain and fatigue. It was thought that these features were simply secondary to the peripheral manifestations of the disease. However, the above findings suggest the BBB may be more porous in RA (Nishioku et al., 2011)and that intracerebral TNFα activity has been demonstrated and (Thomson et al., 2014), thus, opens the possibility of a primary, cerebral etiology for neuropsychiatric symptoms as proposed by Rech et al. (2013).

The influence of infections in triggering and then maintaining a pro-inflammatory response in RA is not fully understood. Laboratory-based studies have focused on the well-characterized pro-inflammatory response induced by agents such as LPS, which is clearly specific to gram negative bacteria. However, the risk of infection for patients on biologics for RA can also be influenced by patient characteristics including age, disease activity, leukopoenia, with co-morbidities including dementia, chronic lung disease, alcoholism and diabetes mellitus, baseline steroid use and female sex. An early systematic review of risk of serious infections in 5014 patients with RA taking anti-TNF therapy (infliximab and adalimumab with etanercept excluded due to differences in structure) in placebo-controlled trials found that serious infections requiring anti-microbial therapy or hospitalization occurred in 126 patients in the treatment arm and 26 patients on placebo (Bongartz et al., 2006). This demonstrated an increased risk of infection with an odds ratio of 2.0, which changed to 2.3 when stratified for dose (Bongartz et al., 2006). Similar findings have been found in other studies with a 2.7–2.8-fold increase in serious infections associated with infliximab and etanercept treatment (Listing et al., 2005) and an incidence rate of 1.79 with subcutaneous abatacept (Alten et al., 2014).

The most recent systematic review at the time of writing was an analysis of data to inform an update of EULAR recommendations for biologic therapies. This review concluded that most articles referring to serious infection demonstrated a significantly increased risk of infection in those treated with anti-TNF therapy when compared to conventional DMARD treatment (adjusted hazard ratio 1.0–1.7) (Ramiro et al., 2014).

A meta-analysis and simple pooling of data from 18 studies (8808 subjects) was used by Leombruno and colleagues to investigate the safety of TNF inhibitor therapy in RA (Leombruno et al., 2009). Etanercept was included in the analysis as the investigators considered that similarities out-weighed the differences between the agents. In this study there was no significant difference in the rate of serious infections between patients on anti-TNF treatment and those not. The mean duration of therapy was less than 1 year, therefore the authors recommended caution in drawing conclusions with regard to long-term safety of biologics. However, a later systematic review investigating the safety of further biologic treatment after initial anti-TNF failure found that serious infection rates were not significantly different to placebo (although only four trials met the eligibility criteria for the review) (Schoels et al., 2012), lending weight to the findings of Leombruno et al. (2009). A further meta-analysis of 12 randomized controlled trials investigating the risk of serious infections during rituximab, abatacept, and anakinra treatment for RA found no increased risk of infections for abatacept, rituximab or low-dose anakinra but an increased risk for high-dose anakinra (OR 3.4) (Salliot et al., 2009).

The risk of infection for patients on biologic therapy is affected by the other medications used to treat the condition. A study by Germano and colleagues demonstrated that, compared to the incidence ratio of infection in patients taking conventional DMARDS and corticosteroids, those taking anti-TNF and DMARDS had a two-fold increase in incidence ratio, and those taking anti-TNF and corticosteroids had a three-fold increase in incidence ratio (Germano et al., 2014). This dropped to a 2.5-fold increase with anti-TNF, corticosteroid and DMARD suggesting that corticosteroids increase the risk of infection when used with anti-TNF. Infection risk (though not serious infection risk) whilst taking adalimumab has been shown to increase with increasing dose of concurrent methotrexate therapy in biologic and DMARD naïve patients (Burmester et al., 2014). The risk of infections appears to vary depending on the biologic agent, with studies showing that opportunistic infections were less common when using etanercept compared to the monoclonal agents infliximab (OR 17.6) and adalimumab (OR 10.0) (Salmon-Ceron et al., 2011).

## Timing of infections in the context of anti-TNF or immunosuppressant treatment

Animal models have shown the peak of reported effects in the brain to occur within a few hours following the acute phase response (Serrats and Sawchenko, 2009). Peripheral LPS injection in mice has been shown to elicit Interferon Stimulating Genes (ISG) induction between 6 and 48 h after exposure (Thomson et al., 2014). However, Thomson and colleagues found that the ISG up regulation was not wholly TNF dependent, as shown by the lack of up regulation of all ISGs with TNFα alone (Thomson et al., 2014). The same group showed that IL-1beta and TNF alpha were both independently up regulated in the brain following multiple LPS challenges (Thomson et al., 2014). Nadeau and Rivest (1999) have also suggested that LPS can have rapid effects on the hypothalamic-pituitary-adrenal axis was determined by measuring the transcriptional activity of corticotropin-releasing factor and plasma corticosterone levels. It is therefore conceivable that clinical RA disease activity, which can flare after acute infection, may also be linked to neuro-inflammation during episodes of clinical infection.

Askling and Dixon (2008) found that infection risk was highest in the first few months of anti-TNF treatment, and then declined in frequency. This effect was echoed in another study demonstrating that the risk of serious infection was greater at 12 weeks duration of TNF inhibitor therapy (odds ratio 2.08) than at 104 weeks duration (odds ratio 0.97). This finding may be due to a shift in the drug safety profile of TNF inhibitor agents, with persistent blockade of the TNF pathway leading to up-regulation of an alternative immune pathway and thus a compensation of immunity (Rosenblum and Amital, 2011). However, the decline in infection risk with time may be due to confounding factors. As the active RA is controlled by the anti-TNF agent, the dose of immunosuppressant CS is reduced, leading to a possible recovery of host immunity. It is also hypothesized that some patients may be at an inherently higher risk of contracting infections. If they are included within the biologic treatment arm of a randomized controlled trial they may develop an infection soon after starting therapy, the drug will then be stopped and they will leave the cohort leaving a healthier cohort of patients. The latter is known as the “healthy user effect” (Rosenblum and Amital, 2011).

In terms of opportunistic infections (excluding TB), the median time from commencement of treatment to time of first opportunistic infection is 16.2 months (6.0–26.0) (Salmon-Ceron et al., 2011), much longer than the 3 months of highest risk, suggesting that immunosuppression secondary to biologics is, in fact, delayed in onset. It should be noted that studies since that performed by Dixon and colleagues (Askling and Dixon, 2008) have shown that the risk of first serious infection was stable throughout treatment.

Numerous murine and human models have demonstrated how LPS-induced gram-negative infections induce inflammation accompanied by somatic or visceral pain (Teeling et al., 2012; Chiu et al., 2013; Thomson et al., 2014). Such features have been attributed to sensitisation of nociceptors by inflammatory mediators released by immune cells. Sensitisation of nociceptors by inflammatory mediators released by peripheral immune cells occurs through activation of the TLR4 signaling pathway by LPS a toxic product of bacterial lysis. Meseguer et al. (2014) recently proposed that LPS induces activation of TRPA1 in a murine model, a transient receptor potential cation channel that trasduces environmental stimuli to nociceptor activity. The group also found that acute vascular reactions, including neurogenic inflammation as indicated by CGRP release (measured by enzyme immunoassay), were mediated through TRPA1 and were independent of TLR4 activation. Such observations suggest that not only are infectious agents such as LPS inducing the pain response, but also produce a neurovascular response which could explain the missing link between peripheral infection and central brain neuro-inflammation.

## Neuro-immune interaction in rheumatoid arthritis

Neuroinflammation is conducted, in the CNS, by microglial cells and astrocytes through the production of pro-inflammatory mediators and cytokines. This inflammation results in cell death and neuronal loss. RA is characterized by chronic inflammation which leads to a constant stimulation of nociceptor and excitation of afferent neurons to the brain. As such it has neurological similarities to a chronic pain state and central sensitization is thought to occur in as a result (Woolf, 1983). Evidence for enhanced hyperalgesia has been demonstrated in RA patients when compared to controls using a capsaicin and pin-prick method (Morris et al., 1997).

Lee and colleagues have suggested central sensitization as a mechanism for explaining chronic pain in rheumatic diseases such as RA (Lee et al., 2011). Central sensitization and changes in nociceptive processing in response to repeated stimulation by chemo-somatosensory event-related potentials (CSSERP) have been demonstrated encephalographically in RA patients (Wendler et al., 2001). Although increases in alpha_1_ and beta_1_ range are thought to be secondary to a chronic pain state it may be that these changes are in fact a result of direct neuroinflammation secondary to the arthritis itself. Schweinhardt et al. found that people with painful RA demonstrate higher activation of brain regions associated with emotional cognition of pain including the medial pre-frontal cortex (Schweinhardt et al., 2008).

Further evidence of neuro-immune interplay has also emerged suggesting that use of biologic therapies such as TNF inhibitors can have central brain effects in people with RA well before a reduction of inflammation within affected joints occurs. In a recent study functional MRI (fMRI) was used to assess the neurological effects of TNF inhibitor therapy in 10 patients with active RA (Rech et al., 2013). The fMRI signal is sensitive to concentration of deoxyhemoglobin (which is paramagnetic), hence can detect areas of higher neuronal activity where there is greater blood flow due to increased brain metabolism associated with a task or stimulus (Ogawa et al., 1990). Rech et al. (2013) analyzed the fMRI response to compression of finger joints in RA patients at baseline and 3, 7, and 28 days after subcutaneous treatment with the TNF inhibitor adalimumab. Clinical disease activity was assessed at the same time points by applying the validated DAS28 (Disease Activity Score), which is based on the assessment of the number of swollen and tender joints, the ESR and the patient's global rating of disease activity. Inflammatory tissue was also measured anatomically by MRI of the dominantly affected hand at baseline and 28 days. At baseline there was a greater volume of functional activation in response to finger joint compression of the patients that responded to TNF treatment than those that did not respond. The main regions activated by joint compression at baseline were the somatosensory cortex, insular cortex and dorsolateral prefrontal cortex, thus suggesting a greater perception of pain. However, the brain activation was then significantly reduced as early as 3 days after treatment and further decreased after 7 days, whereas the disease activity score was only significantly reduced by day 28. Data from this study support the concept that chronic inflammation in RA leads to central sensitization, with the possibility of modulating pain perception with appropriate treatment.

A neuro-immune interaction is not isolated to RA or TNFα. Neuropsychiatric symptoms have shown improvement with cytokine blockade, for example IL-1 inhibition in Sjögrens and diabetes mellitus reduced fatigue (Norheim et al., 2012) Within “pure” neuroinflammatory disorders such as Alzheimer's there is increasing murine data to show an improvement in memory and reduced hippocampal TNFα levels as a result of anti-TNFα receptor fusion protein administration (Detrait et al., 2014). This has lead to a move toward considering anti-TNFα as a therapy for Alzheimer's dementia (Cheng et al., 2014) Auanofin, a gold—containing medication and established treatment for RA, dampens inflammation through manipulation of the anti- and pro-inflammatory interleukin balance. Interestingly a recent *in vitro* study has demonstrated a reduced production of cytotoxic mediators by microglia in response to gold therapy (Medeiros et al., 2007).

Diamond and Tracey recently proposed the concept of the immunological homunculus of the brain (Diamond and Tracey, 2011) where the brain acts as a sensory organ, allowing real-time transmission of information such as infections, tissue damage and inflammation to the central nervous system. Indeed, they draw attention to the concept of an “inflammatory reflex,” in which the neurotransmitter acetyl choline interacts with cytokine production via α7 nicotinic acetylcholine receptors on immune cells.

## Magnetic resonance imaging in rheumatoid arthritis

Magnetic resonance imaging (MRI) provides a variety of techniques for assessing neurological involvement of disease processes by detecting changes in brain morphometry [T1 weighted imaging (T1w)] and tissue microstructure [T2 weighted imaging (T2w), diffusion weighted imaging (DWI), diffusion tensor imaging (DTI), and magnetisation transfer imaging (MTI)], brain metabolism by magnetic resonance spectroscopy (MRS) and actual brain functionality by fMRI as described above. However, there are far fewer reports of neuroimaging abnormalities for RA compared to other related autoimmune diseases. In Sjögren's syndrome significant loss of brain tissue and presence of white matter lesions are observed (Akasbi et al., 2012; Lauvsnes et al., 2014).Lupus can lead to microstructural changes in white matter (WM), white matter lesions and metabolic abnormalities (Axford et al., 2001; Zivadinov et al., 2013) with associated neuropsychological sequelae. A summary of the imaging techniques utilized so far in demonstrating inflammatory changes in autoimmune-mediated conditions is summarized in Table 1.

**Table 1:** **A table displaying the imaging modalities used to investigate neuroinflammation**.

**Imaging modality**	**Method of action**	**Relevance to rheumatoid arthritis**
T2-Weighted MRI	Inflammatory and degenerative changes are demonstrated in the form of white matter hyperintensities	White matter lesions are associated with higher levels of S100B which is a potential surrogate marker for blood brain barrier disruption (Hamed et al., 2012)
T1-Weighted MRI	Provides high spatial resolution imaging of the brain allowing volumetric assessment of cerebral anatomy	Has demonstrated increased volume of basal ganglia in RA patients compared to controls (Steens et al., 2005)
Diffusion weighted imaging	Quantitative parameters relate to neuronal density, structural integrity and water content	Potential for assessing inflammatory and neurodegenerative changes (Steens et al., 2005; Zivadinov et al., 2013)
Magnetisation transfer imaging	Quantitative parameters relate to magnetisation exchange between macromolecules (e.g., myelin, proteins) and bulk tissue water	Potential for assessing inflammatory and neurodegenerative changes (Steens et al., 2005)
Magnetic resonance spectroscopy	Relative levels of specific metabolites, including N-acetyl aspartate, creatine, choline, myo-Inositol and some neurotransmitters	Increased choline/creatine ratio is proportional to rise in ESR and disease activity Increased choline levels seen in neuroinflammation in systemic lupus (Axford et al., 2001; Emmer et al., 2009)

T2 weighted imaging allows detection of lesions associated with inflammatory and degenerative changes in the brain and in terms of number and size of WM hyperintensities, lesion load is higher with age in the normal population and associated with microvascular disease (King et al., 2014). A quantitative T2w MRI study showed no significant difference in WM lesion load between RA patients and controls (Bekkelund et al., 1995) although WM lesions in RA patients have been associated with higher levels of the protein S100B, a potential plasma marker of BBB disruption and neurodegenerative effects (Hamed et al., 2012). Hamed and colleagues performed cognitive testing and found that RA patients had greater depression than controls, but this did not correlate with cognitive scores, whereas cognition did correlate with S100B levels (Hamed et al., 2012). In a rodent model of arthritis the presence of the inflammation stimulated protein S100A4 was strongly associated with BBB disruption (Nishioku et al., 2011). Thus a cerebrovascular component is likely in RA, but not one specifically detectable by conventional MRI in the general RA population.

T1 weighted imaging provides high spatial resolution of the brain with excellent contrast between gray and white matter structures to allow assessment of the shape and size of different anatomical regions. A recent study with T1w MRI demonstrated no global difference of intracranial volume between RA patients and controls, but observed significant increases in the volume of basal ganglia structures (Wartolowska et al., 2012). The caudate nucleus in particular showed the most significant increase, suggesting these changes are related to pain processing rather than the disease itself. However, there are reports of both increased and decreased gray matter structure volumes in patients with chronic pain indicating there may be a complex mix of neurodegenerative or adaptive changes dependent on disease, duration and consequent lifestyle changes, as well as treatment (Wartolowska et al., 2012). Currently it is known that only in patients with longstanding (i.e., >15 years) RA is there a reduction in brain volume that may relate to neurodegenerative changes caused by RA (Bekkelund et al., 1995).

Treatment for RA commonly includes CS to reduce inflammation and the effect of low-dose CS treatment on the brain has been investigated with quantitative MRI (Steens et al., 2005). Parameters derived from DWI and MTI relate to neuronal density and myelin-tissue water magnetisation exchange respectively, hence are sensitive to inflammatory and neurodegenerative changes. Whole brain histograms of the apparent diffusion coefficient (ADC) and magnetization transfer ratio (MTR) showed no significant difference between controls and RA patients with or without CS treatment. Although the results of Steens et al. (2005) indicate no global differences of MRI parameters, a more focused study that separates gray and white matter structures is warranted as performed for lupus and with the increased sensitivity of modern 3T MRI systems (Zivadinov et al., 2013). In addition, DTI has much greater sensitivity than DWI to detect microstructural changes in WM, and can provide detailed anatomical maps that describe regional differences of neurodegenerative change (Barrick et al., 2010).

There is some evidence of metabolic changes in the brain due to RA. In the Steens study (Steens et al., 2005) 1H MRS of a single white matter region adjacent to the left ventricle almost reached a significant difference in metabolite levels between RA patients and controls. A subsequent retrospective analysis showed that a high choline to creatine (Cho/Cr) metabolite ratio was associated with high ESR levels and correlated with ESR and disease activity after correction for disease activity and duration (Emmer et al., 2009). There was no difference in N-acetyl aspartate to creatine (NAA/Cr) ratio or correlation with ESR, suggesting there was no neuronal damage, which is consistent with the lack of major neurological symptoms in RA, unlike for lupus. Elevated choline is found in lupus and other neuroinflammatory diseases (Axford et al., 2001). Choline has a function in cell membrane synthesis and its elevation in RA may relate to microglial activation or monocyte infiltration (Emmer et al., 2009). Brain metabolic changes have been associated with pain processing regions in a number of studies and elevation of the combined neurotransmitters glutamate and glutamine observed in fibromyalgia patients (Fayed et al., 2010) and also with induced pain (Gussew et al., 2010). The significance of these results for development of novel treatment or monitoring treatment response is yet to be established.

To date there are far fewer neuroimaging studies in RA patients compared to other diseases with cerebrovascular, neuroinflammatory and neurodegenerative components (e.g., stroke, lupus and dementias). This reflects the lower incidence of neurological symptoms and the more subtle effects of cerebrovascular involvement that have been so far detected.

## Conclusion

The concept of neuroinflammation as a significant component of disease pathophysiology in RA has only recently been recognized. Animal models have recently demonstrated that infectious agents including LPS can directly activate the immune system stimulate pain responses. Coupled with the strong link between RA and incidence of infection, it may be time to reconsider the balance between maintaining disease remission using disease-modifying immune-modulatory drugs and preventing disease flares induced by infection. However, the link between infection and immune-modulatory therapies remains controversial and comparison of studies is made difficult by differing biologic therapies used, different diseases treated and different, concurrent medications regimes. Further, high-powered studies are required to establish the period of highest risk and susceptibility to infections and the overall risk of infections for patients taking biological treatments for RA.

Future work is needed to understand how the RA disease process impacts on pain activation in RA, whether sensitisation is a general phenomenon in RA or found in specific subsets of patients and what specific infectious triggers contribute to an autoimmune pro-inflammatory network that leads to chronic pain and inflammation.

### Conflict of interest statement

The authors declare that the research was conducted in the absence of any commercial or financial relationships that could be construed as a potential conflict of interest.

## Abbreviations

ACPA: Anti-Citrullinated Protein Antibody
AD: Alzheimer's Disease
BBB: blood-brain barrier
Cho: Choline
Cr: Creatine
COX: Cyclo-Oxygenase
CS: Corticosteroids
CSSERP: Chemo-Somatosensory Event-Related Potentials
DAMPs: damage associated molecular patterns
DMARD: Disease-Modifying Anti-Rheumatic Drug
DRG: Dorsal Root Ganglia
DTI: Diffusion Tensor Imaging
ESR: Erythrocyte Sedimentation Rate
fMRI: Functional Magnetic Resonance Imaging
ICAM: intercellular adhesion molecule
IFN-β: Interferon-β
IL: Interleukin
ISG: Interferon-Stimulating Genes
LPS: lipopolysaccharide
MRI: Magnetic Resonance Imaging
MRS: Magnetic Resonance Spectroscopy
NAA: N-Acetyl Aspartate
PADIV: Peptidyl Arginine Deaminase type IV
RA: Rheumatoid Arthritis
RNA: ribonucleic acid
SE: Shared Epitope
SNP: Single Nucleotide Polymorphism
TACE: Tumor necrosis factor alpha converting enzyme
Th: T helper cell
TLR: Toll-like receptor
TNFα: tumor necrosis factor alpha
WM: White Matter.
